# Feeding Recovery in Post-PICU Patients: A Case Series in an Intensive Feeding Program

**DOI:** 10.3390/nu18081291

**Published:** 2026-04-20

**Authors:** Tariq Almanaseer, Ellen Hayhurst, Jessica B. Doorn, Ashley Bonebrake, Brooke Dudick, Elizabeth A. Rosner, Nancy F. Bandstra, Mara L. Leimanis-Laurens

**Affiliations:** 1 College of Human Medicine, Michigan State University, Grand Rapids, MI 49503, USA; tariqzeyadmohammad.almanaseer@corewellhealth.org (T.A.); hayhurs5@msu.edu (E.H.); 2 Pediatric Intensive Care Unit, Helen DeVos Children’s Hospital, Corewell Health, Grand Rapids, MI 49503, USA; jessica.doorn@corewellhealth.org (J.B.D.); ashley.jousma@corewellhealth.org (A.B.); elizabeth.rosner@helendevoschildrens.org (E.A.R.); 3 Corewell Health Research Institute, Corewell Health, Grand Rapids, MI 49503, USA; brooke.dudick@corewellhealth.org; 4 Pediatrics and Human Development, College of Human Medicine, Michigan State University, East Lansing, MI 48824, USA; 5 Intensive Feeding Program, Helen DeVos Children’s Hospital, Corewell Health, Grand Rapids, MI 49503, USA; nancy.bandstra@helendevoschildrens.org; 6 Departments of Psychiatry and Pediatrics & Human Development, Michigan State University, East Lansing, MI 48824, USA

**Keywords:** critical care, nutrition, PICU, PICS-p, intensive feeding program, interdisciplinary behavioral treatment

## Abstract

Background/Objectives: Survival after pediatric intensive care unit (PICU) admission has improved, yet many children experience post-intensive care syndrome in pediatrics (PICS-p), including persistent feeding difficulties that impair growth and quality of life. An intensive feeding program (IFP), also known as intensive interdisciplinary behavioral treatment (IIBT), reduces tube dependence and improves oral intake; however, outcomes in PICU survivors remain understudied. This study aimed to evaluate feeding outcomes in children with prior PICU admission who completed IIBT. Methods: This study was a retrospective case series of children (0–18 years) admitted to the HDVCH, Corewell Health, Grand Rapids, Michigan, who subsequently completed IIBT (from 2007 to 2024). Variables included demographics, PICU course (admission indication, complications, length of stay, ventilation, and nutrition status) and IIBT outcomes (feeding modality, oral skills, and malnutrition status). Feeding outcomes were compared pre- and post-IIBT. Results: Sixteen patients were included (62.5% female; mean age 1.44 ± 1.21 years). Primary PICU admission causes were post-operative recovery (68.8%) and acute respiratory failure (25%). PICU complications included acute respiratory failure (43.8%) and the need for respiratory support beyond baseline (62.5%). At PICU discharge, 75% remained tube-fed and 18.8% were malnourished. The mean time from PICU discharge to IIBT initiation was 641 ± 385 days. At IIBT baseline, 75% were tube-fed and all were non-self-feeders. Following IIBT completion (mean length of stay 4.8 ± 0.9 weeks), 58% of tube-fed patients achieved tube removal eligibility; 44% transitioned to partial or full self-feeding; problematic mealtime behaviors decreased (45.7% → 9.9%); oral acceptance improved (62% → 95%); and mouth clearance improved (59% → 96%). Malnutrition prevalence decreased (20% → 12%). Conclusions: Children with prior PICU admission demonstrated substantial feeding and behavioral improvement during IIBT participation, with over half achieving tube-weaning eligibility. The time from referral to program start reflects barriers that delay intervention.

## 1. Introduction

Advances in medical care within pediatric intensive care units (PICUs) have led to significant improvements in survival rates for critically ill children [1,2]. As a result, there is an increasing focus on the long-term consequences of intensive care and the morbidity experienced by pediatric patients after PICU discharge [3,4,5,6]. The term post-intensive care syndrome in pediatrics (PICS-p) has been used to describe the range of new or worsening impairments in physical, cognitive, emotional, and social health that may persist following a PICU stay and are particularly relevant to pediatric development and family dynamics [1,2,3,4,5]. These impairments can negatively impact a child’s ability to achieve normal function and development, affecting their quality of life and placing additional burden on families and caregivers [1,2,3,4,5].

Nutritional outcomes and feeding recovery serve as important indicators of how the physical, emotional, and social impact of intensive care can unfold for children and families. Among the potential long-term morbidities following PICU admission, feeding difficulties are of significant concern, particularly in young children [7]. PICU admissions often involve multiple physical and environmental stressors, including negative and painful oral experiences such as endotracheal intubation, extubation, re-intubation, and nasogastric tube (NGT) insertion [1,8]. Such procedures can result in unpleasant symptoms relating to feeding intolerance and dysphagia, including vomiting, diarrhea, and reflux [9,10]. It is common for children in the PICU to not eat or drink orally, with NGT feeding frequently used for nutritional support [11]. Prolonged NGT feeding can negatively impact the development of normal oral feeding experiences and may contribute to subsequent feeding difficulties post-discharge [4,5,12]. These feeding issues can manifest as limited oral intake, poor growth, lack of progression to table food, restrictive food preferences, feeding aversion, and, in the most severe cases, oral aversion, where children exhibit distress in response to even non-nutritive oral stimulation [7,13].

In addition to feeding difficulties, malnutrition is a well-recognized concern among PICU patients, with both pre-existing undernutrition and acute illness-related malnutrition contributing to impaired recovery [14]. Critical illness increases metabolic demands while simultaneously limiting oral intake, placing children at high risk for nutrient deficits that may worsen during hospitalization and prolong recovery trajectories [15,16]. These concerns are especially important in children with underlying feeding challenges. Children requiring mechanical ventilation, whether invasive (IMV) or non-invasive (NIV), may also experience persistent respiratory difficulties and fatigue post-PICU, potentially impacting their feeding behaviors and abilities [17]. Feeding difficulties post-PICU can lead to poor nutritional intake, delayed growth, and increased parental anxiety and stress, and may impede overall recovery and quality of life for children [1,4,7]. Given the prevalence of feeding and nutritional challenges among PICU survivors, targeted interventions that support oral intake and nutritional recovery play an important role in supporting long-term recovery.

Intensive interdisciplinary behavioral treatment (IIBT) has been shown to address feeding difficulties in various pediatric populations, including those with oral aversion [8,18]. Sustained progress in feeding recovery is strengthened by the structured training and support for patients and families provided by IIBT. However, these programs remain a limited resource nationally. A 2024 report identified 16 such programs in the United States, only one of which is located in Michigan [19]. These programs often involve a multidisciplinary approach, incorporating medicine, psychology, nutrition, speech-language pathology, and occupational therapy to target oral-motor feeding skills, desensitization to oral stimulation, and improved oral nutrition [8]. Behavioral interventions based on conditioning paradigms are frequently used in IIBT to improve compliance and advance feeding therapy [8]. Research suggests that IIBT can lead to significant improvements in acceptance of oral intake and mealtime behaviors, reduced parental stress, and reductions in tube dependence in children with feeding difficulties [13,20].

For children with a past admission to the PICU, proper nutrition is especially important to support proper growth and recovery from severe illness. Evidence supports individualized nutritional protocols from an established nutritional team to reduce morbidity in critically ill children in the ICU [21]. While the literature acknowledges the prevalence of feeding problems after PICU discharge and the potential benefits of IIBT for children with feeding disorders while in the PICU, there is a need for more research specifically examining the effects of IIBT on clinical outcomes in children with a history of a PICU stay. Understanding the impact of IIBT on this population can help inform best practices for post-PICU care, optimize nutritional recovery, and potentially decrease long-term effects of critical illness.

This study aims to address this gap by examining a case series of feeding outcomes of patients with a prior PICU admission who subsequently underwent IIBT to remediate feeding problems. This study intends to investigate retrospective data from patients’ stay in the PICU in combination with their outcomes from the IIBT to identify potential modifiable risk factors to success in the IIBT. Long-term outcome data in this patient population is sparse, as it requires long-term follow-up, convergent data sets of electronic medical records (EMRs), broad IRB coverage, and a cross-disciplinary study team involving dieticians, pediatric intensivists, critical care data managers, clinical researchers, and clinical psychologists. Our goal is to better understand the feeding difficulties associated with PICU admission, as has been previously explored in our hospital setting with multiple patient etiologies, as well as explore any associations between PICU-related factors and treatment response [22,23,24,25,26].

## 2. Materials and Methods

### 2.1. Nutritional Assessment

#### Determination of Nutrition Goals

Nutrition-related variables included daily caloric and protein intake while in the PICU, extracted from diet orders and intake/output flowsheets. Nutritional prescriptions were based on Dietary Reference Intake (DRI) or resting energy expenditure (REE) calculations by age and gender, or on home nutrition plans for patients receiving baseline enteral feeds. Enteral versus parenteral feeding route, tube type (nasogastric—NG, nasojejunal—NJ, gastrostomy—GT, or gastro-jejunal—GJ), water intake route, and primary feeding modality (oral vs. tube) were documented. Feeding at discharge was also recorded, including transitions to new tube types, increased tube dependence, new oral restrictions, or initiation of total parenteral nutrition (TPN). Malnutrition was classified at PICU admission and discharge according to American Society for Parenteral and Enteral Nutrition (ASPEN) pediatric criteria (normal, mild, moderate, or severe).

### 2.2. Study Design

#### 2.2.1. Site and Patient Population of HDVCH

**Figure 1:**
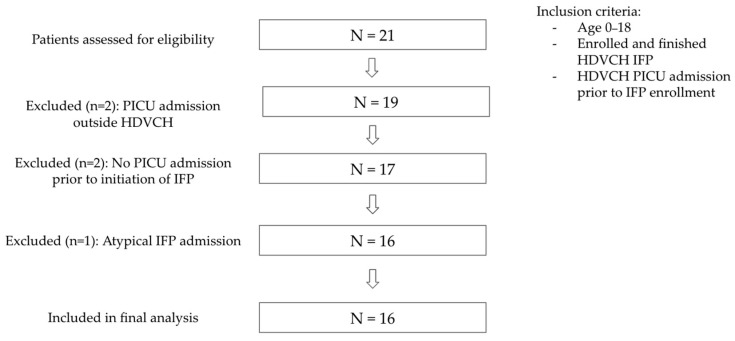
Flow diagram of patient inclusion and exclusion in study.

This study employed a retrospective case series design of 16 pediatric patients (0–18 years) with a documented history of PICU admission at a high-volume, urban tertiary facility in Western Michigan who also completed an IIBT from 2007 to 2024. An initial population of 21 patients meeting these criteria was identified. Inclusion criteria were updated to require that the most recent PICU admission prior to starting the IIBT was at our facility. This PICU admission was then used for data collection. Five patients were subsequently excluded due to the most recent PICU admission before IIBT being outside of our facility, PICU admission after beginning the IIBT, and an atypical IIBT admission, resulting in a total of 16 patients to be used in data analysis (Figure 1). Data from patients’ most recent PICU admission and hospitalization course prior to start of the IIBT was paired with data from their subsequent IIBT course. Feeding outcomes were compared pre- and post-IIBT.

The study and data collection were conducted at Helen DeVos Children’s Hospital (HDVCH), Corewell Health (CH) located in Grand Rapids, Michigan, United States. HDVCH pediatric critical care unit (PCCU) has more than 1500 admissions per year with more than 6000 patient-days. Seventeen board-certified intensivists cover the 24-bed unit with the flexibility and capability to care for up to 36 critically ill patients.

#### 2.2.2. Site and Patient Population of Intensive Feeding Program

The HDVCH CH IFP is an ambulatory clinic that provides IIBT to treat pediatric feeding disorders. Services are provided by members of an interdisciplinary treatment team, including medicine, psychology, nutrition, speech-language pathology, occupational therapy, medical social work, child life, and culinary science. IIBT is offered via an intensive outpatient model, providing three therapeutic feedings every weekday across a 6- to 8-week admission. Therapeutic feedings are completed by a speech-language pathologist, occupational therapist, or certified occupational therapy assistant trained in a behavioral feeding protocol created and modified by psychologists. As part of a child’s admission, parents/caregivers are trained in the established protocol to promote generalization of treatment gains and ongoing progression in the home setting. Post-discharge, children are followed for a year during intermittent visits with the treatment team.

The study was conducted according to the guidelines of the Declaration of Helsinki and approved by the Institutional Review Board (or Ethics Committee) of Corewell Health (protocol code: IRB 2014-103 SH/HDVCH). Patient consent was waived due to the nature of this minimal-risk retrospective chart review. Parent consent was obtained for the case example.

### 2.3. Data Extraction, Management, and Quality Control

#### 2.3.1. HDVCH CH

A comprehensive set of variables was collected for all included patients. General demographic descriptors obtained from the local EMR included age, sex, race, ethnicity, height, weight, and body mass index (BMI). PICU-related variables consisted of the number of prior PICU admissions, dates of the most recent admission and discharge prior to initiating IIBT, length of stay (days), severity of illness on admission using the Pediatric Risk of Mortality (PRISM) score, PICU admission diagnosis, and type of clinical complications during PICU admission. The PRISM score is a 17-point variable scoring system measured over the first 24 h of admission. Variables were classified as binary, ordinal, integer, and numeric.

Respiratory support variables included whether support exceeded baseline needs, the type of support received (IMV, NIV, pressure-based support, high-flow nasal cannula (HFNC), or low-flow oxygen therapy), duration of invasive ventilation prior to extubation or tracheostomy placement, duration of non-invasive support, new dependence on respiratory support at discharge, extracorporeal membrane oxygenation (ECMO) utilization, and total ECMO duration (days). For patients with tracheostomies, additional support requirements and time to return to baseline were recorded, along with discharge ventilator support type.

Manual data extraction included PICU admission dates and length of stay, admission indications, complications, respiratory support characteristics, and feeding-route details. Nutritional calculations and malnutrition classifications were completed by a team of dietitians.

#### 2.3.2. Intensive Feeding Program

Data related to the IIBT included dates of program start and completion, length of stay (days and weeks), baseline and discharge feeding modality (oral, tube, or TPN), feeding status (exclusive non-self-feeder, exclusive self-feeder, or transitional), and behavioral feeding metrics—mealtime acceptance (i.e., the percentage of trials completed within 5 s), mouth clearance (i.e., the percentage of bites/drinks swallowed within 30 s), and problematic mealtime behaviors (i.e., the percentage of trials in which negative and/or problematic mealtime behaviors were observed)—collected at baseline (prior to treatment) and during the final treatment week. For patients with feeding tubes, tube-removal eligibility, timing of tube removal, and oral intake percentage at baseline and program completion were recorded. Malnutrition status was similarly assessed at IIBT start and conclusion using ASPEN criteria.

Data from the IIBT, including dates of program start and completion, baseline and discharge feeding modality and status, and behavioral feeding metrics were populated from the previously IRB-approved Research Registry in the HDVCH Corewell Health IFP for the purpose of performing retrospective research studies. Automatically extracted data included demographic descriptors.

All data for both data sets were populated using Research Electronic Data Capture (REDCap^®^). Quality control for manual data extraction by two independent reviewers of variables regarding PICU admission was determined based on the inter-rater reliability (found to be 97.4% for 117 data points [~17% of total PICU admission data set]). This exceeded the predetermined threshold for repeating data collection (85%); therefore, re-extraction was not required.

### 2.4. Data Analysis

#### Outcome Measures

Outcome measures included the most recent PICU length of stay; the number of PICU admissions before starting IFP; PICU complications including respiratory failure, hypertension, heart failure, arrhythmia, adrenal insufficiency, pneumonia, cardiac arrest, fluid overload, requirement of respiratory support, duration of respiratory support, and requirement of ECMO; changes in feeding characteristics pre/post-PICU including tube feeding, tube type, and malnutrition status; changes in feeding characteristics from IIBT baseline to discharge including feeding type, feeding status, and malnutrition status; and changes in mealtime performance metrics from IIBT baseline to discharge including rate of acceptance and mouth clearance, problematic mealtime behaviors, and oral intake and IIBT calorie and protein intake characteristics including daily goals, daily averages, and proportion of days meeting goals.

Categorical data were expressed as count (%). Numeric data were expressed as mean ± standard deviation or median (25th and 75th percentile) depending on the normality of the data. Normality was assessed using Shapiro–Wilk normality tests. Mealtime performance metric differences within subjects from baseline to IFP discharge were tested using paired *t*-tests and significance was assessed at α = 0.05.

## 3. Results

### 3.1. Patient Inclusion, Demographics, PICU Admissions and Complications

A total of 16 patients with a history of PICU admission who subsequently completed the IFP were included in the analysis (Figure 1). Given that PICU admission data collection was based on the most recent admission prior to starting the IIBT, two patients were excluded due to their most recent PICU admission prior to beginning the IIBT being outside of our facility. Two patients were excluded due to a lack of PICU admission before starting the IIBT, while one patient was excluded due to an atypical IIBT course (treatment goals and/or progression were not comparable to the broader patient group).

The mean age at PICU admission was 1.44 ± 1.21 years, and 10 patients (62.5%) were female. Most patients were White (68.8%), with smaller proportions identifying as Black or African American (12.5%), Asian (6.3%), or other races (12.5%). One patient (6.3%) was of Hispanic ethnicity. PICU admissions reflected a heterogeneous group of critical illnesses, and many patients had more than one contributing diagnosis. The most common reasons for PICU admission were post-operative recovery from non-cardiac surgery (43.8%), acute respiratory failure (25.0%), and post-cardiac surgery care (25.0%), with smaller numbers admitted for severe infection, dehydration, renal causes, and other cardiac conditions (Table 1). Additional clinical details are included in Supplementary Table S1.

PICU complications were frequent. Nearly half of the patients developed acute respiratory failure during admission (43.8%), and 62.5% required respiratory support beyond their baseline needs. Overall, 31.3% required IMV (median duration 1 day [1, 7]), and 50.0% received non-invasive respiratory support (median duration 2 days [1, 3]). Additional complications included heart failure requiring medication (25.0%), fluid overload requiring medication (18.8%), hypotension requiring vasoactive support (18.8%), arrhythmias requiring intervention (12.5%), and isolated cases of hypertension requiring infusion therapy, adrenal insufficiency, pneumonia, and cardiac arrest. No patients required ECMO (Table 2).

Patients had a median of two PICU admissions before starting the intensive feeding program [1, 3.5]. The median length of stay for the most recent PICU admission was 4.5 days [1, 7], and the median maximum PRISM score was 2.5 [0, 3.5], reflecting generally low-to-moderate illness severity in this cohort.

### 3.2. PICU Feeding Characteristics

At baseline in the PICU, 12 of 16 patients (75.0%) were tube-fed, most commonly via a GT (58.3% of tube-fed patients), followed by GJ, NG, and NJ tubes. Of tube-dependent patients, 12 of 12 were tube-fed from birth due to swallowing difficulties (including aspiration, gagging, vomiting, and coughing), extreme prematurity, or multiple surgeries after birth requiring nil per os (NPO) status. Five patients (31.3%) had documented oral feeding at PICU baseline (not mutually exclusive with tube feeding). Most patients were classified as normally nourished at PICU baseline (87.5%), while 6.3% had mild and 6.3% had moderate malnutrition. By PICU discharge, the proportion of patients receiving tube feeding (75.0%) and oral feeding (31.3%) remained similar to baseline, and only one patient (6.3%) had a documented change in feeding modality compared with PICU baseline. Nutritional status at discharge was generally stable: 81.3% were classified as normally nourished, 12.5% had mild malnutrition, and 6.3% had moderate malnutrition, with only one patient demonstrating a worsening in malnutrition status between PICU admission and discharge (Table 3).

### 3.3. Post-PICU: IFP Referral, Evaluation and Intake

**Figure 2:**
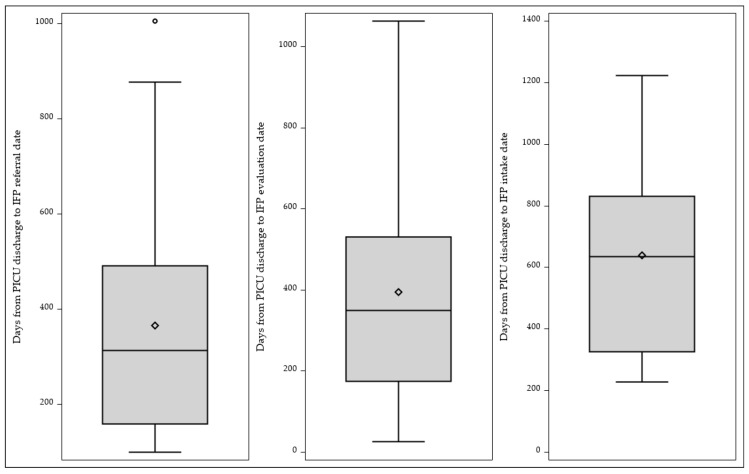
Days from PICU discharge to IFP (IIBT) (N = 16); ◊ represents the mean value.

Among the 16 patients included in the analysis, substantial delays were observed between discharge from the PICU and subsequent steps in accessing intensive feeding services. The median time from PICU discharge to referral was 312.5 days (interquartile range [IQR], 158.5–491.0 days). Additional delays were noted between PICU discharge and initial evaluation, with a median of 349.5 days (IQR, 174.0–531.5 days). The longest interval occurred between PICU discharge and program intake, with a median duration of 634.5 days (IQR, 325.0–832.0 days), indicating that many patients experienced delays of more than one year before initiating intensive feeding intervention. Once enrolled, patients remained in the program for a mean of 33.3 ± 6.1 days, corresponding to 4.8 ± 0.9 weeks of intensive daily treatment (Figure 2).

### 3.4. Post-PICU: IFP Outcomes of Feeding Type, Status and Malnutrition

At IIBT baseline, 12 patients (75.0%) were tube-dependent and four (25.0%) were taking some oral feeds. All patients were classified as exclusive non-self-feeders (100%), reflecting a high level of caregiver dependence for feeding at program entry. Most patients were classified as normally nourished at IIBT baseline (80.0%), with mild malnutrition documented in 20.0%; malnutrition data were missing for one patient.

Mealtime performance measures indicated substantial feeding difficulties at baseline. The mean rate of acceptance of offered bites was 62.2% ± 21.4%, and mean mouth clearance was 59.4% ± 28.9%. Problematic mealtime behaviors were common, occurring during nearly half of meal opportunities (45.7% ± 18.8%). Among tube-dependent patients, oral intake accounted for an average of only 4.8% ± 9.6% of total nutrition at baseline.

By program discharge, feeding outcomes and behaviors showed marked improvement. Among the 12 patients who were tube-fed at IIBT baseline, seven (58.3%) met predefined criteria for tube removal for feeding purposes. Overall feeding status also shifted toward greater independence: nine patients (56.3%) remained exclusive non-self-feeders, while three (18.8%) became exclusive self-feeders and four (25.0%) transitioned to partial self-feeding (Table 4).

### 3.5. Post-PICU: IFP Outcomes of Mealtime Performance

**Figure 3:**
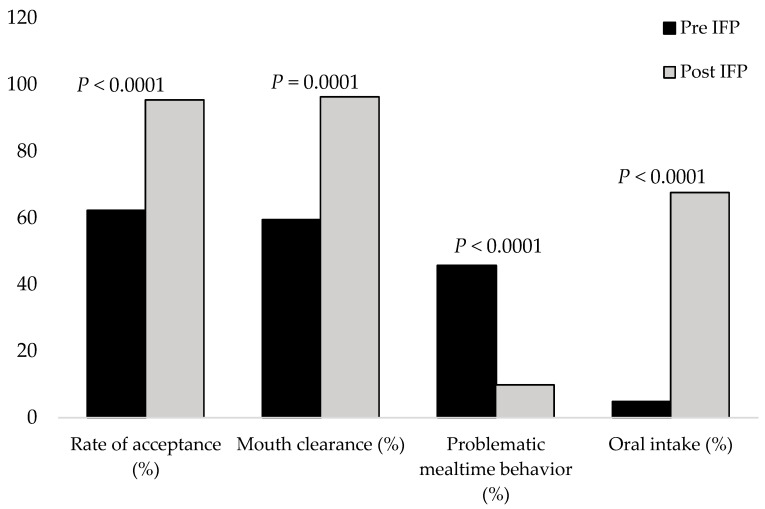
The figure visualizes the improvement in feeding performance from baseline to discharge with mean ± SD. IFP: intensive feeding program.

Mealtime performance metrics improved across all domains. The mean rate of acceptance increased to 95.4% ± 6.0%, and mean mouth clearance rose to 96.3% ± 5.6% at discharge. Problematic mealtime behaviors decreased to 9.9% ± 7.6%, representing a substantial reduction in disruptive behaviors during feeding. Among initially tube-dependent patients, the proportion of nutrition taken orally increased to a mean of 67.6% ± 24.9% by program completion (Figure 3).

Nutritional status at IIBT discharge remained favorable: 87.5% of patients were classified as normally nourished, and 12.5% had mild malnutrition, with no patients classified as moderately or severely malnourished.

### 3.6. PICU: Caloric and Protein Intake

Detailed caloric and protein intake data were available for a subset of 10 patients, 9 of whom had individualized daily caloric and protein goals. Only patients who were in the PICU for >24 h had their daily intake recorded due to the gap between PICU admission and referral to nutrition support with subsequent full intake and data collection. On average, patients met their daily calorie goals on 16% ± 17% of days and their daily protein goals on 58% ± 35% of days (range 0–100%). The proportions can be translated as percentages. For example, 0.35 = 35% (Table 5).

### 3.7. Case Example

“Elizabeth”, an alias (fake name), is a spunky little girl with a complex medical history significant for congenital nephrotic syndrome and end-stage renal disease, ultimately resulting in a kidney transplant at 2 years and 2 months of age. Following the transplant, she remained in the hospital for approximately 3 weeks, the first 3 days of which were spent in the PICU. Elizabeth’s PICU stay was complicated by a difficult re-intubation post-transplant (due to fluid overload), resulting in cardiac arrest on day 3 and subsequent resuscitation. Elizabeth was extubated to high-flow oxygen on the following day and subsequently weaned from further respiratory support.

Regarding her feeding, Elizabeth was exclusively GT tube-fed prior to and during her time in the PICU. Prior to her PICU admission, she was completing six bolus tube feedings daily. Feeding intolerance and vomiting were observed at that time, believed to be related to the severity of her renal failure. Following the transplant, Elizabeth resumed tube feeding in the PICU via a continuous schedule. She transferred out of the PICU on a continuous schedule 8 days following her transplant.

At the time of her intake into the feeding program, Elizabeth was 3 years and 4 months of age. Elizabeth presented with an extended history of significant vomiting related to her kidney disease and suspected dairy intolerance. While she had accepted at least partial feedings via a bottle early in her life, Elizabeth had been primarily tube-fed since birth. Prior to her intake in the program, periods of oral intake were minimal and limited to, at most, small bites of puree and sips of flavored water. Oral feedings were challenging, with Elizabeth consistently crying, pushing and turning away from the utensil and gagging. Upon admission to the program, Elizabeth was 100% tube-fed. Bolus feedings via the G-tube were tolerated comfortably.

At the start of treatment, Elizabeth was placed on a behavioral feeding protocol without food or drink present. She was initially presented with non-nutritive expectations (e.g., dry spoon trials) during three daily therapeutic sessions, targeting intra-oral desensitization. Within her first two treatment weeks, liquid (initially water) and then food (pureed applesauce, initially diluted with water) were introduced by spoon. Over the course of the next several treatment weeks, a nutritious drink and additional purees were introduced during her therapeutic mealtimes. Except for periods of illness and the introduction of dairy, which triggered gagging and vomiting and was thus discontinued, Elizabeth tolerated the introduction of new foods well, showing high acceptance and minimal negative behaviors. Parents completed training in her established feeding protocol during her seventh and eighth treatment weeks, successfully generalizing her oral feedings to the home setting without significant difficulty.

Elizabeth was discharged after 39 days of treatment. During her final treatment week, Elizabeth demonstrated high rates of acceptance (m = 94%) and low rates of problematic mealtime behaviors (m = 15%). She was discharged obtaining 79% of her calories by mouth. By her 2-week post-discharge follow-up, Elizabeth had progressed further in her oral intake, resulting in complete tube-weaning (for calories, though not hydration given her nephrological status) by that time.

## 4. Discussion

In this retrospective case series of children with prior PICU admission who completed an intensive interdisciplinary behavioral feeding program, we observed substantial improvements in feeding performance, feeding modality, and mealtime behaviors following treatment. Despite prolonged intervals between PICU discharge and initiation of specialized feeding intervention, participation in the IFP was associated with marked gains in oral intake, reduced problematic mealtime behaviors, and meaningful progression toward feeding independence, including tube-weaning eligibility in more than half of tube-dependent patients. These findings suggest that intensive feeding intervention can be effective even when delivered long after the acute critical illness and highlight feeding recovery as an important, potentially modifiable domain of post-intensive care syndrome in pediatrics (PICS-p).

### 4.1. Feeding Recovery as a Component of PICS-p

PICS-p encompasses a broad range of physical, cognitive, emotional, and social impairments that may persist following critical illness [27,28]. While much of the existing literature has focused on neurodevelopmental, functional, and psychosocial outcomes, feeding difficulties remain an under-recognized but clinically significant manifestation of PICS-p up to two and four years after a PICU admission [29,30]. In this cohort, most patients with prior tube dependence experienced continued tube dependence and significant feeding dysfunction that persisted well beyond PICU discharge, suggesting an association between critical illness, PICU admission and associated interventions, and feeding development [31]. These findings align with prior work demonstrating that PICU survivors, particularly young children, are at increased risk for feeding aversion, oral-motor delay, and maladaptive mealtime behaviors following exposure to intubation, tube feeding, and prolonged hospitalization [32].

Importantly, our results demonstrate that feeding dysfunction in PICU survivors is not necessarily static or irreversible. Participation in an intensive interdisciplinary feeding program was associated with dramatic improvements across objective behavioral metrics, including acceptance, mouth clearance, and problematic mealtime behaviors, as well as meaningful clinical outcomes such as increased oral intake and eligibility for tube removal. These improvements support the conceptualization of feeding recovery as a dynamic component of post-PICU rehabilitation that may respond to targeted, structured intervention.

### 4.2. Impact of Intensive Interdisciplinary Feeding Treatment

The magnitude of improvement observed across feeding performance metrics in this cohort is consistent with prior studies evaluating intensive interdisciplinary behavioral treatment in children with feeding disorders, including those with oral aversion and tube dependence [8,13,18,20]. However, few studies to date have specifically examined outcomes in children with a history of PICU admission. Our findings extend the existing literature by demonstrating that children recovering from critical illness—despite heterogeneous diagnoses, multiple PICU admissions, and prolonged delays to intervention—can achieve substantial gains with intensive feeding therapy.

Notably, more than half of tube-dependent patients were determined by the HDVCH CH IFP to have the possibility of living without a feeding tube following program completion, and oral intake among tube-fed patients increased from minimal levels at baseline to more than two-thirds of total nutritional intake on average at discharge. Improvements in feeding independence were also observed, with a substantial proportion of patients transitioning from exclusive non-self-feeding to partial or full self-feeding. These outcomes suggest that IFPs may play a critical role in mitigating long-term feeding morbidity among PICU survivors and reducing prolonged reliance on enteral feeding.

### 4.3. Timing of Intervention and Delayed Referral

A striking finding in this study was the prolonged delay between PICU discharge and initiation of intensive feeding treatment, with a mean interval of nearly two years. This delay likely reflects multiple systemic and patient-level barriers, including limited availability of specialized feeding programs, delayed recognition of feeding dysfunction as a post-PICU morbidity, competing medical priorities, and challenges related to care coordination following hospital discharge [33]. Other possibilities for delay include patients’ medical, nutritional, and/or developmental readiness for intensive interdisciplinary feeding treatment [33]. Despite this delay, patients demonstrated robust improvements with intervention, suggesting that feeding recovery remains possible even when treatment is initiated well after the acute illness phase.

Nevertheless, earlier identification and referral may offer additional benefits. Delays in addressing feeding dysfunction may allow maladaptive behaviors to become entrenched, prolong caregiver stress, and increase the risk of nutritional compromise or growth failure. These findings highlight the need for increased awareness of feeding outcomes among PICU survivors and support the incorporation of feeding assessment into post-PICU follow-up and survivorship programs.

### 4.4. PICU-Related Factors and Feeding Outcomes

Although this study was not powered to detect definitive associations between specific PICU-related variables and feeding outcomes, the cohort exhibited a high prevalence of respiratory complications and respiratory support beyond baseline needs. The prior literature has suggested that invasive and non-invasive ventilation, prolonged respiratory support, and respiratory fatigue may contribute to feeding difficulties following critical illness [7,17]. The heterogeneous nature of PICU diagnoses and generally low-to-moderate illness severity in this cohort may have limited the ability to identify clear predictors of feeding recovery. Nonetheless, the observed feeding impairments across patients with varied PICU courses reinforce the notion that feeding dysfunction may arise across a broad spectrum of critical illness experiences, rather than being confined to the most severe cases.

Due to the retrospective study design and small sample size, improvements in feeding outcomes cannot be attributed definitively to IIBT. Pediatric feeding disorders are inherently multifactorial, involving medical, nutritional, and social domains, making it difficult to isolate the effect of any single intervention on feeding outcomes. A recent meta-analysis found that while current tube-weaning interventions are generally effective, there exist considerable differences in treatment approaches, suggesting heterogeneity in drivers of treatment success [34]. Improvements may also reflect the natural course of feeding recovery, as some children transition from tube to oral feeding with the improvement of medical conditions or progression through developmental stages. Additional factors, including medical complexity, developmental readiness, and caregiver influence, likely contribute and were not accounted for in this analysis, offering areas of future investigation [34,35].

### 4.5. Limitations

The retrospective design and small sample size do not imply causal inference and generalizability. While this study shows significant improvement in the pre- and post-intervention comparison, the authors cannot with absolute certainly claim to what extent the effects of spontaneous recovery and the passage of time may have contributed to patient outcomes. The absence of a control group precludes comparison with PICU survivors who did not undergo intensive feeding treatment. Feeding intake and nutritional data during the PICU stay were incomplete for some patients, and long-term follow-up beyond program completion was not available.

The patient population lacks racial diversity, as has been previously reported in cohort studies from our group of 68% [23] and 72% [36] for Caucasians and is consistent with state census data [37]. The IFP does not report race as a mandatory field in their data collection. Missing data for the IFP has been previously reported [13] as 25%; a strategy to compensate could include future studies with hospital admissions outside of the PICU to increase sample size and with rehabilitation centers (such as Mary Freebed).

Case scenarios were explored for this case series. Due to limitations and logistical constraints around the consenting process, one family gratefully revealed their care journey and treatment trajectory for our wider readership. Permission was offered to the senior author and their care provider (NB) after careful consideration from all participants.

### 4.6. Future Directions

Future research should aim to prospectively evaluate feeding outcomes in PICU survivors, identify early predictors of persistent feeding dysfunction, and determine optimal timing for intervention. Multicenter studies with larger sample sizes are needed to better understand how illness severity, respiratory support, nutritional practices, and psychosocial factors interact to influence feeding recovery. Incorporating feeding assessments into post-PICU follow-up clinics may facilitate earlier identification of feeding difficulties and improve access to targeted interventions.

## 5. Conclusions

In this case series of PICU survivors with feeding dysfunction, participation in an intensive interdisciplinary behavioral feeding program was associated with substantial improvements in feeding performance, oral intake, and feeding independence, even when intervention occurred long after PICU discharge. These findings support feeding recovery as an important and modifiable component of PICS-p and highlight the potential role of intensive feeding programs in improving long-term outcomes for children recovering from critical illness.

## Figures and Tables

**Table 1:** Patient population demographics and admission diagnoses.

Variable	Whole Sample (N = 16)
Sex	
Female	10 (62.5)
Male	6 (37.5)
Race	
White or Caucasian	11 (68.8)
Black or African American	2 (12.5)
Asian	1 (6.3)
Other	2 (12.5)
Hispanic ethnicity	1 (6.3)
Age (Years)	1.44 ± 1.21
Admission diagnosis	
Acute respiratory failure	4 (25.0)
Severe infection	1 (6.3)
Cardiac condition	2 (12.5)
Post-operative recovery (non-cardiac)	7 (43.8)
Severe dehydration	1 (6.3)
Renal causes	1 (6.3)
Cardiac surgery, post-operative	4 (25.0)
Number of PICU admissions prior to IFP	2 [1, 3.5]
Length of stay of most recent PICU admission (days)	4.5 [1, 7]
Maximum PRISM score during PICU stay	2.5 [0, 3.5]

**Table 2:** PICU admission complications.

Variable	Whole Sample (N = 16)
Acute respiratory failure	7 (43.8)
Hypotension requiring vasopressor support	3 (18.8)
Hypertension requiring continuous infusion	1 (6.3)
Heart failure requiring pharmacologic therapy	4 (25.0)
Arrhythmia requiring intervention	2 (12.5)
Adrenal insufficiency requiring stress-dose steroids	1 (6.3)
Pneumonia	1 (6.3)
Cardiac arrest	1 (6.3)
Fluid overload requiring medication	3 (18.8)
Required respiratory support beyond baseline	10 (62.5)
Required invasive respiratory support	5 (31.3)
Duration of invasive ventilation (days)	1 [1, 7]
Required non-invasive respiratory support	8 (50.0)
Duration of non-invasive ventilation (days)	2 [1, 3]
Type of non-invasive respiratory support	
HFNC	3 (37.5)
Nasal cannula or face mask	5 (62.5)
Tracheostomy with increased respiratory support from baseline	2 (12.5)
Duration of increased respiratory support (days)	2.5 [1, 4]
ECMO required	0 (0)

Complications during the most recent PICU admission among the cohort (N = 16), with acute respiratory failure being the most common complication (n = 7), followed by heart failure requiring medications (n = 4) and hemodynamic instability requiring vasopressor support (n = 3). HFNC: high-flow nasal cannula.

**Table 3:** PICU feeding characteristics—baseline and discharge.

Variable	Baseline (N = 16)	Discharge (N = 16)
Tube feeding	12 (75.0)	12 (75.0)
Tube type		
Nasojejunal (NJ)	1 (8.3)	—
Nasogastric (NG)	2 (16.7)	—
Gastrostomy tube (GT)	7 (58.3)	—
Gastrojejunostomy tube (GJ)	2 (16.7)	—
Oral feeding	5 (31.3)	5 (31.3)
Malnutrition status		
Normal nutrition	14 (87.5)	13 (81.3)
Mild malnutrition	1 (6.3)	2 (12.5)
Moderate malnutrition	1 (6.3)	1 (6.3)
Change in feeding modality (oral vs. tube) from baseline to discharge	—	1 (6.3)
Change in malnutrition status from baseline to discharge	—	1 (6.3)

**Table 4:** IFP feeding characteristics—baseline and discharge.

Variable	Baseline (N = 16)	Discharge (N = 16)
Feeding Type		
Oral feed	4 (25.00)	—
Tube feed	12 (75.00)	—
Eligibility for tube removal	—	7 (58.33) (N = 12 eligible)
Feeding Status		
Exclusive non-self-feeders (NSFs)	16 (100)	9 (56.25)
Exclusive self-feeders (SFs)	0 (0)	3 (18.75)
Transitional self-feeders (Trans)	0 (0)	4 (25.00)
Malnutrition Status		
Normal nutrition	12 (80.00)	14 (87.50)
Mild malnutrition	3 (20.00)	2 (12.50)

**Table 5:** Caloric and protein intake during PICU hospitalization.

Variable	Sample (N = 10)
Calorie daily goal (kilocalories)	N = 9
Mean ± SD	854.4 ± 359.2
Min–Max	352–1300
Protein daily goal (grams of protein)	N = 9
Mean ± SD	10.5 ± 3.1
Min–Max	6.2–14.6
Calorie daily average (kilocalories)	
Mean ± SD	519.1 ± 302.1
Min–Max	172.55–1157
Protein daily average (grams of protein)	
Mean ± SD	15.4 ± 12.0
Min–Max	1.2–41.2
Proportion of days meeting goal calories	N = 9
Mean ± SD	0.16 ± 0.17
Min–Max	0–0.45
Proportion of days meeting goal protein	N = 9
Mean ± SD	0.58 ± 0.35
Min–Max	0–1.00 ^1^

^1^ Note: Calorie and protein data is only included for whole days (all days between first and last day); 10 patients were included, and 9 of those 10 patients had a daily calorie and protein goal.

## Data Availability

Inquiries regarding data contributions can be directed to the corresponding author.

## Supplementary Materials

The following supporting information can be downloaded at: https://www.mdpi.com/article/10.3390/nu18081291/s1, Table S1: PICU Patients reasons for admissions and clinical backgrounds.

## Abbreviations

ADEM: acute disseminated encephalomyelitis; ARDS: acute respiratory distress syndrome; ASPEN: American Society for Parenteral and Enteral Nutrition; BMI: body mass index; CRRT: continuous renal replacement therapy; DI: diabetes insipidus; DIC: disseminated intravascular coagulopathy; DRI: dietary reference intake; ECMO: extracorporeal membrane oxygenation; EMR: electronic medical record; GJ: gastro-jejunal tube; GT: gastrostomy tube; HFNC: high-flow nasal cannula; ICU: intensive care unit; IFP: intensive feeding program; IIBT: intensive interdisciplinary behavioral treatment; IMV: invasive mechanical ventilation; IQR: interquartile range; NG: nasogastric tube; NGT: nasogastric tube; NIV: non-invasive ventilation; NJ: nasojejunal tube; NPO: nothing by mouth; NSF: non-self-feeder; PICU: pediatric intensive care unit; PICS-p: post-intensive care syndrome in pediatrics; PRISM: Pediatric Risk of Mortality Score; REE: resting energy expenditure; SF: self-feeder; SIADH: syndrome of inappropriate diuretic hormone; TPN: total parenteral nutrition.

## References

[1] Ekim A. (2020). The Post-Intensive Care Syndrome in Children. Compr. Child Adolesc. Nurs..

[2] Quadir A., Festa M., Gilchrist M., Thompson K., Pride N., Basu S. (2024). Long-term follow-up in pediatric intensive care—A narrative review. Front. Pediatr..

[3] Manning J.C., Pinto N.P., Rennick J.E., Colville G., Curley M.A.Q. (2018). Conceptualizing Post Intensive Care Syndrome in Children-The PICS-p Framework. Pediatr. Crit. Care Med..

[4] Rahmaty Z., Manning J.C., Perez M.H., Ramelet A.S. (2023). Post Intensive Care Syndrome in Swiss Paediatric survivors and their Families (PICSS-PF): A national, multicentre, longitudinal study protocol. BMJ Open.

[5] Curley M.A.Q., Watson R.S., Killien E.Y., Kalvas L.B., Perry-Eaddy M.A., Cassidy A.M., Miller E.B., Talukder M., Manning J.C., Pinto N.P. (2024). Design and rationale of the Post-Intensive Care Syndrome—Paediatrics (PICS-p) Longitudinal Cohort Study. BMJ Open.

[6] Rennick J.E., Childerhose J.E. (2015). Redefining success in the PICU: New patient populations shift targets of care. Pediatrics.

[7] Morton K., Marino L.V., Pappachan J.V., Darlington A.S. (2019). Feeding difficulties in young paediatric intensive care survivors: A scoping review. Clin. Nutr. ESPEN.

[8] Morton K., Darlington A.E., Marino L.V. (2020). Protocol for a multicentre longitudinal mixed-methods study: Feeding and survivorship outcomes in previously healthy young paediatric Intensive care survivors (the PIES Study). BMJ Open.

[9] Hoffmeister J., Zaborek N., Thibeault S.L. (2019). Postextubation Dysphagia in Pediatric Populations: Incidence, Risk Factors, and Outcomes. J. Pediatr..

[10] Li Y., Fu C.H., Ju M.J., Liu J., Yang X.Y., Xu T.T. (2024). Measurements of enteral feeding intolerance in critically ill children: A scoping review. Front. Pediatr..

[11] Mehta N.M., Skillman H.E., Irving S.Y., Coss-Bu J.A., Vermilyea S., Farrington E.A., McKeever L., Hall A.M., Goday P.S., Braunschweig C. (2017). Guidelines for the Provision and Assessment of Nutrition Support Therapy in the Pediatric Critically Ill Patient: Society of Critical Care Medicine and American Society for Parenteral and Enteral Nutrition. J. Parenter. Enter. Nutr..

[12] Gottrand F., Sullivan P.B. (2010). Gastrostomy tube feeding: When to start, what to feed and how to stop. Eur. J. Clin. Nutr..

[13] Bandstra N.F., Huston P.L., Zvonek K., Heinz C., Piccione E. (2020). Outcomes for Feeding Tube-Dependent Children With Oral Aversion in an Intensive Interdisciplinary Treatment Program. J. Speech Lang. Hear. Res..

[14] Abera E.G., Sime H. (2023). The prevalence of malnutrition among critically ill children: A systematic review and meta-analysis. BMC Pediatr..

[15] Agrawal A., Sharma S., Janjua D., Jadon G., Chanchlani R., Dsouza V. (2025). Impact of nutritional status on the mortality and clinical outcomes of children admitted to the pediatric intensive care unit: A systematic review and meta-analysis. Clin. Nutr..

[16] Kratochvil M., Klucka J., Klabusayova E., Musilova T., Vafek V., Skrisovska T., Djakow J., Havrankova P., Osinova D., Stourac P. (2022). Nutrition in Pediatric Intensive Care: A Narrative Review. Children.

[17] MacDonald S., Du Pont-Thibodeau G., Thibault C., Jutras C., Roumeliotis N., Farrell C., Ducharme-Crevier L. (2024). Outcomes of patients supported by mechanical ventilation and their families two months after discharge from pediatric intensive care unit. Front. Pediatr..

[18] Seiverling L., Hendy H.M., Yusupova S., Kaczor A., Panora J., Rodriguez J. (2020). Improvements in Children’s Feeding Behavior after Intensive Interdisciplinary Behavioral Treatment: Comparisons by Developmental and Medical Status. Behav. Modif..

[19] Sharp W.G., Malugen E., Pederson J., Martin-Halpine L., Dempster R., Baranwal N., Hodges A., Raol N., Volkert V.M. (2024). Intensive Multidisciplinary Feeding Day Programs in the United States: A Report Regarding the Treatment Landscape. J. Pediatr..

[20] Sharp W.G., Volkert V.M., Scahill L., McCracken C.E., McElhanon B. (2017). A Systematic Review and Meta-Analysis of Intensive Multidisciplinary Intervention for Pediatric Feeding Disorders: How Standard Is the Standard of Care?. J. Pediatr..

[21] Briassoulis G., Ilia S., Briassouli E. (2024). Personalized Nutrition in the Pediatric ICU: Steering the Shift from Acute Stress to Metabolic Recovery and Rehabilitation. Nutrients.

[22] Brackmann M., Lintvedt A., Kogelschatz B., Heinze E., Parker J.L., Ferguson K., Rosner E., Boville B., Leimanis-Laurens M.L. (2023). Daily Nutritional Intake of Pediatric Patients (N = 64) on Extracorporeal Membrane Oxygenation from 2018 to 2022: A Single-Center Report. Nutrients.

[23] Leimanis Laurens M.L., Jaji A.M., Montgomery J., Jess J., Ferguson K., Parker J., Sanfilippo D., Rajasekaran S. (2020). Preadmission Diet and Zip Code Influences the Pediatric Critical Care Clinical Course for Infants with Severe Respiratory Illness (N = 187). J. Pediatr. Intensive Care.

[24] Leimanis-Laurens M.L., Ferguson K., Wolfrum E., Boville B., Sanfilippo D., Lydic T.A., Prokop J.W., Rajasekaran S. (2021). Pediatric Multi-Organ Dysfunction Syndrome: Analysis by an Untargeted “Shotgun” Lipidomic Approach Reveals Low-Abundance Plasma Phospholipids and Dynamic Recovery over 8-Day Period, a Single-Center Observational Study. Nutrients.

[25] Lintvedt A., Purosky I., Kogelschatz B., Brackmann M., Heinze E., Parker J., Dudick B., McDiarmid J., Rosner E., Boville B. (2024). Nutritional Intake in Venovenous ECMO Patients: A Single-Center Study in a North American PICU. Nutrients.

[26] Russell M.M., Leimanis-Laurens M.L., Bu S., Kinney G.A., Teoh S.T., McKee R.L., Ferguson K., Winters J.W., Lunt S.Y., Prokop J.W. (2022). Loss of Health Promoting Bacteria in the Gastrointestinal Microbiome of PICU Infants with Bronchiolitis: A Single-Center Feasibility Study. Children.

[27] Fink E.L., Maddux A.B., Pinto N., Sorenson S., Notterman D., Dean J.M., Carcillo J.A., Berg R.A., Zuppa A., Pollack M.M. (2020). A Core Outcome Set for Pediatric Critical Care. Crit. Care Med..

[28] Maddux A.B., Pinto N., Fink E.L., Hartman M.E., Nett S., Biagas K., Killien E.Y., Dervan L.A., Christie L.M., Luckett P.M. (2020). Postdischarge Outcome Domains in Pediatric Critical Care and the Instruments Used to Evaluate Them: A Scoping Review. Crit. Care Med..

[29] Verlinden I., Güiza F., Dulfer K., Van Cleemput H., Wouters P.J., Guerra G.G., Joosten K.F., Verbruggen S.C., Vanhorebeek I., Van den Berghe G. (2022). Physical, Emotional/Behavioral, and Neurocognitive Developmental Outcomes From 2 to 4 Years After PICU Admission: A Secondary Analysis of the Early Versus Late Parenteral Nutrition Randomized Controlled Trial Cohort. Pediatr. Crit. Care Med..

[30] Jacobs A., Dulfer K., Eveleens R.D., Hordijk J., Van Cleemput H., Verlinden I., Wouters P.J., Mebis L., Guerra G.G., Joosten K. (2020). Long-term developmental effect of withholding parenteral nutrition in paediatric intensive care units: A 4-year follow-up of the PEPaNIC randomised controlled trial. Lancet Child Adolesc. Health.

[31] Hopwood N., Moraby K., Dadich A., Gowans J., Pointon K., Ierardo A., Reilly C., Syrmis M., Frederiksen N., Disher-Quill K. (2021). Paediatric tube-feeding: An agenda for care improvement and research. J. Paediatr. Child Health.

[32] Winderlich J., Little B., Oberender F., Bollard T., Farrell T., Jenkins S., Landorf E., McCall A., Menzies J., O’Brien K. (2025). Nutrition support in children discharged from the pediatric intensive care unit: A bi-national prospective cohort study (ePICUre). J. Pediatr. Gastroenterol. Nutr..

[33] Baranwal N., Hodges A., Breiner C.E., Malugen E., Estrem H.H., Sharp W.G., Raol N. (2024). Intensive Outpatient Programs for Pediatric Feeding Disorder: A Qualitative Study of Current Challenges and Future Directions. J. Dev. Behav. Pediatr. JDBP.

[34] Killian H.J., Bakula D.M., Wallisch A., Swinburn Romine R., Fleming K., Edwards S.T., Bruce A.S., Chang C.N., Mousa H., Davis A.M. (2023). Pediatric Tube Weaning: A Meta-Analysis of Factors Contributing to Success. J. Clin. Psychol. Med. Settings.

[35] Lively E.J., McAllister S., Doeltgen S.H. (2019). Variables Impacting the Time Taken to Wean Children From Enteral Tube Feeding to Oral Intake. J. Pediatr. Gastroenterol. Nutr..

[36] Kampfschulte A., Oram M., Escobar Vasco A.M., Essenmacher B., Herbig A., Behere A., Leimanis-Laurens M.L., Rajasekaran S. (2021). Understanding Suicide in Our Community through the Lens of the Pediatric ICU: An Epidemiological Review (2011–2017) of One Midwestern City in the US. Children.

[37] United States Census Bureau QuickFacts Michigan. https://www.census.gov/quickfacts/fact/table/MI/PST045224.

