# Supplementary material for: Feeding Recovery in Post-PICU Patients: A Case Series in an Intensive Feeding Program
Source: Nutrients. 2026 Apr 20;18(8):1291. doi: 10.3390/nu18081291 (PMC13118444; doi:10.3390/nu18081291)
Supplement: Supplementary file 1 [file nutrients-18-01291-s001.zip › nutrients-4220124-supplementary.pdf]

Table-S1: PICU Patients reasons for admissions and clinical backgrounds

Patient	1° dx	2° dx	Age	Gender	PRISMIII	Complications
1	Cardiac surgery, post-op		2-6	F	2	Heart failure requiring medications
2	Post-op recovery (non-cardiac)		0-2	M	3	No major complications
3	Severe dehydration	Renal insufficiency	0-2	M	3	No major complications
4	Post-op recovery (non-cardiac)		0-2	M	0	No major complications
5	Post-op recovery (non-cardiac)		0-2	M	0	No major complications
6	Cardiac surgery, post-op	Cardiac condition	0-2	M	4	Acute respiratory failure<br>Hypotension requiring pressors<br>Heart failure requiring medications<br>Arrhythmia requiring intervention<br>Fluid overload requiring medications
7	Cardiac surgery, post-op		0-2	F	5	Acute respiratory failure<br>Heart failure requiring medications<br>Arrhythmia requiring intervention
8	Acute respiratory failure		2-6	F	0	Acute respiratory failure
9	Post-op recovery (non-cardiac)		0-2	F	3	No major complications
10	Acute respiratory failure	Severe infection	0-2	F	0	No major complications
11	Post-op recovery (non-cardiac)		0-2	M	3	Acute respiratory failure
12	Cardiac surgery, post-op	Cardiac condition	0-2	M	5	Acute respiratory failure<br>Hypotension requiring pressors<br>Heart failure requiring medications<br>Fluid overload requiring medications
13	Acute respiratory failure		0-2	M	1	Pneumonia
14	Acute respiratory failure		0-2	M	0	No major complications
15	Post-op recovery (non-cardiac)		0-2	F	8	Acute respiratory failure<br>Hypotension requiring pressor<br>Adrenal insufficiency requiring steroids<br>Cardiac arrest<br>Fluid overload requiring medications
16	Post-op recovery (non-cardiac)		2-6	M	0	No major complications

Notes: Age in years.
